# Demographic Factors and Job Characteristics Associated With Burnout in Chinese Female Nurses During Controlled COVID-19 Period: A Cross-Sectional Study

**DOI:** 10.3389/fpubh.2021.757113

**Published:** 2022-01-06

**Authors:** Li-Li Zhou, Shu-E Zhang, Jiao Liu, Hong-Ni Wang, Li Liu, Jing-Jing Zhou, Zhi-Hua Bu, Yu-Fang Gao, Tao Sun, Bei Liu

**Affiliations:** ^1^Department of Urology, The Second Affiliated Hospital, Harbin Medical University, Harbin, China; ^2^Department of Health Management, School of Health Management, Harbin Medical University, Harbin, China; ^3^Department of Respiratory Medicine, Third Affiliated Hospital, Harbin Medical University, Harbin, China; ^4^Department of Health Policy and Management, School of Public Health, Hangzhou Normal University, Hangzhou, China; ^5^Department of Nephrology, Second Affiliated Hospital of Harbin Medical University, Harbin, China; ^6^Department of Endocrinology, Second Affiliated Hospital of Harbin Medical University, Harbin, China; ^7^Institute of Hospital Management, Qingdao University, Qingdao, China; ^8^Department of Inspection, School of Public Health, Peking University, Beijing, China

**Keywords:** female nurses, burnout, factors, a cross-section, controlled COVID-19 period

## Abstract

**Background:** To investigate the prevalence of burnout syndrome among Chinese female nurses during the controlled coronavirus disease 2019 (COVID-19) period and explore its associated socio-demographic factors and job characteristics.

**Methods:** With the multistage, stratified sampling method, a cross-sectional online survey was conducted from September to October 2020 in China. The survey tool included revised Maslach Burnout Inventory (MBI) with 15 items, socio-demographic and job characteristics. Univariate logistic regression analysis and multivariate factor logistic regression analysis were used to identify the risk factors for burnout of female nurses.

**Results:** During controlled COVID-19 period in China, the overall prevalence of burnout symptoms among Chinese female nurses was 60.2% with a breakdown in severity as follows: 451 (39.8 %) mild, 163 (14.4%) moderate, and 68 (6.0%) severe burnout. Little variance was reported for burnout symptoms according to job tenure (*Waldχ*^2^ = 14.828, *P* < 0.05,odds ratio [OR] <1), monthly salary income (*Waldχ*^2^ = 12.460, *P* < 0.05, OR <1), and night shift (*Waldχ*^2^ = 3.821, *P* < 0.05, OR > 1).

**Conclusion:** Burnout symptoms among Chinese female nurses were prevalent and associated with job tenure, monthly salary income, and night shift. Female nurses who were with shorter job tenure, worked at night shifts, and had lower monthly salaries tended to exhibit increasing high-level burnout than their counterparts. This study serves as an implication for administrators and policy-makers to improve the work conditions of nurses for promoting overall healthcare service quality.

## Background

Recently, WHO declared burnout as an “occupational phenomenon” in the International Classification of Diseases 11th revision (ICD-11), recognizing burnout as a new public health concern (1). A previous study confirmed (2) that burnout affected the physical and mental health of nurses, quality of nursing care, and conditions and recovery of patients. Burnout is described as a psychological syndrome involving feelings of emotional fatigue, depersonalization (DA), a sense of reduced personal accomplishment (PA), and poor self-efficacy secondary to occupational stress (3, 4). According to Maslach et al., burnout consists of three dimensions involving emotional exhaustion (EE), DA and cynicism, and reduced PA (3). EE refers to the feelings of being overextended emotionally and physically and depleted of personal energy (3). DA means a cynical and distant attitude toward one's duties and tasks. Reduced PA is the tendency to negatively evaluate their own achievements at work (3).

The concept of burnout originated from the field of human service in which professionals contact with other people as the majority of their tasks and which can become a source of stress (5, 6). In recent years, nursing staff regarded as a high-risk and particularly vulnerable population for suffering burnout, are experiencing exceptional challenges worldwide within the current turbulent healthcare system due to these dilemmas, such as limited health resources, staff shortages, overworked, and overloaded (7). Moreover, tense nurse-patient relationships, frequent nurse-patient conflicts, and workplace violence contribute to extensive depressive symptoms and occupational stress for nurses and further resulting in suffering burnout (8). Various studies have been undertaken to evaluate the prevalence of burnout in high-income countries among nurses ranged from 33 to 50% (2, 9–11). The latest evidence found that the prevalence of burnout among nurses has been increasing at an alarming rate in low-income and moderate-income countries, with a previous study reported exceeding 60% in China (12, 13).

Particularity, the coronavirus disease 2019 (COVID-19) outbreak became a public health emergency of international concern (PHEIC) on January 30, 2020 (14). Being the largest part of the healthcare workforce against the epidemic, the hospital industry is facing greater challenges as a result of the increased work stress, highest risk, and heavier workload during the COVID-19 epidemic (15), which potentially contribute to the increased high risk of burnout. However, actions and behaviors of nurses toward the COVID-19 epidemic have attracted intense approval from the public and the media (16), as social support, which may contribute to a positive function related to alleviate the intensive relationship between nurses and patients and increase professional identity and calling of nurses, thereby resulting in decreased nurses burnout. Unfortunately, the limited available literature reported the prevalence of burnout among nurses is still conflicting (17). During the initial and the most severe phase of the COVID-19 epidemic in China, one study reported a high-level prevalence of burnout among Chinese front-line nurses against the epidemic (18). Contrarily, another survey conducted in China showed that nurses who worked in COVID-19 wards had lower burnout symptom than those worked in usual wards (19). The generalizability of findings regarding the prevalence of burnout is inconsistent, since the study was conducted on different stages of the COVID-19 epidemic, and a convenience sample of participating by social media, and varied working demands (20, 21). Therefore, this study believes that the prevalence of burnout of Chinese nurses is of worry and needs to be assessed further during different stages of the COVID-19 epidemic.

Previous studies widely confirmed that burnout was associated with negative attitudes toward work and life (22), such as generating physical symptoms of fatigue, anxiety, and so on, resulting in various forms of job withdrawal behavior and affecting work engagement and quality of nursing care (23) and even fundamental functionality of healthcare systems (24). Given that the mass potentially negative impacts of burnout on both the health and quality of patient care, the driving factors of the rising burnout issue had been paid more attention. Based on previous literature, several demographic and job characteristics were suggested to be associated with burnout among medical staffs, such as age (25), sex (25), job tenure, work hours education level (26), professional titles (27), and so on. However, overall evidence on demographic and job factors of burnout among nurses was inconclusive (28) in different space-time settings and deserves a wide range of attention.

Moreover, the study of gender differences has been historically controversial in previous scientific research (29, 30). Female has changed the role of women in society through progressively entering the workforce and expanding their aspirations in different settings (31). Nursing is a female-subjugated profession with undergoing common work-family conflict and high workload (32). Female nurses are more vulnerable to burnout and psychological distress (33), which tend to cause direct and indirect negative consequences for both patients and themselves. Therefore, it is significant and valuable to explore burnout in female nursing viewpoint, especially in the COVID-19 special period. In relation to burnout specifically, experienced difference between males and female is existent. A meta-analysis showed that females were more likely to be emotionally exhausted than men, whereas males were more likely to report DA than females (29). A previous study drew attention to recognize the differences in burnout between sexes and then required different help and organizational support strategies in dealing with burnout issues (29). In fact, data concerning burnout of female nurse are inconclusive (29). Therefore, considering that gender role interferes with burnout assessment and females are over-represented in disorders, the current study focuses on the prevalence of burnout syndrome of female nurses and tries to accurately identify its association with social-demographic factors and job characteristics. Besides, the COVID-19 period as a special contextual factor is also discussed in the study.

### Aim

The major aims of the present study were to (1) address the prevalence of burnout syndrome among Chinese female nurses during the controlled COVID-19 period and (2) to explore its association with socio-demographic factors and job characteristics.

## Methods

### Subjects and Procedures

We used both multistage-stratified sampling and convenient sampling methods to collect data, a total of 100 cities and 1,740 nurses were randomly selected. The Chinese mainland consists of 32 provinces, which were divided into five regions according to geographical location: eastern, western, southern, northern, and central regions. In each region, 20 cities were randomly selected. An anonymous online survey was completed by a representative group of nurses across 100 cities in 31 provinces in China from September to October 2020 (a controlled COVID-19 period in China). In the second stage of sampling, the nurses who were originally selected were appointed to deliver the questionnaires to other nurses. The selected nurse was invited to click on a webpage link to access a self-administered questionnaire (https://www.wenjuan.com/). Subsequently, the web page link was sent by the deliverers to other nurses via mobile phones. The investigation purpose and significance of this study are shown on the front page of the self-administered questionnaire. The progress of the survey was monitored by the authors. Eventually, we checked the accuracy and completeness of the data and excluded the questionnaire. All data were checked for consistency by the two authors. A total of 1,740 nurses completed their questionnaires, resulting in 1,133 valid questionnaires, with an effective completion rate of 70.13%. The distribution of demographic variables (gender, age, and level of education) of the representative nurses is comparable to the general population of the Chinese registered nurses. The inclusion criteria were recognized as (1) being a registered nurse; (2) being currently enrolled in nursing; (3) gender is female; and (4) consent and voluntary participation in our study.

### Measures

#### Measurement of Demographic Characteristics

In the current study, five demographic variables were collected, such as gender, age, marital status, education level, and parental status. Marital status was divided into three categories: unmarried, married, and divorced or loss of a spouse. In China, one is qualified as a nurse only if who has at least a technical secondary education from a nursing school and has passed Nurse Professional Qualification Examination. Thus, options for educational level included “technical secondary school”, “college degree”, “bachelor's degree”, and “master's degree or above”. Parental status was divided into four categories “no child”, “0–5 years”, “6–18 years”, “19 and above years”.

#### Measurement of Job Characteristics

In the current study, four variables were utilized to assess job characteristics, such as monthly salary income, professional categories, job tenure, and work shift. Professional categories were divided into “nurse”, “senior nurse”, “nurse-in-charge”, and “associate professor of nursing/professor of nursing”. Both “nurse” and “nurse practitioner” are defined as the primary professional title, “nurse-in-charge” as the intermediate professional title, and associate professor of nursing/professor of nursing as the senior professional title in China. Nursing students are graduated from technical secondary nursing school or are graduated from senior high school entrance exam, educational system is divided into 3, 4 years, two kinds of graduates are eligible to become a certified registered nurse after graduation.

The night shift was divided into “yes” or “no”. Monthly income (RMB) was categorized as “3,000 or below”, “3,001–5,000 yuan”, “5,001–7,000 yuan”, “7,001–9,000 yuan”, and “9,001 or above yuan” (1 Yuan ≈ 0.1566 Dollar, 0.1139 Pound, 0.1345 Euro, in October 2021). Using the domestic working-hour standard in China as the cut point, weekly work time was divided into ≤ 40 hours/week and >40 hours/week. Job tenures were divided into categories, such as “1–3”, “4–5”, “6–10”, and “11–20” years. Consider that the employees are more likely to be troubled by stressors (34) even leave their first job (35) in the early career. The demanding and overwhelming experience and work-life interference are important factors influencing new graduate nurse burnout (34, 35). Thus, Relationship between job tenures and burnout is more sensitive to terms. Participants with 1–3 years of job tenures should be considered as new nurses who are a particular group with little working experience, who are prone to have a greater risk of suffering burnout. In comparison, as a similar group with shorter job tenure, participants with 4–5 working experiences should have possessed vocational adaptability.

#### Measurement of Burnout

The MBI (6) with 15 revised items was used to measure the three dimensions of burnout (EE, DA, and reduced PA) translated by Li et al. (36). Items were scored on a seven-point Likert scale ranging from 0 (totally disagree) to 6 (totally agree) where higher scores represented a higher degree of burnout. This burnout inventory in medical settings was demonstrated to have good reliability and validity in the Chinese context. The Chinese version of Maslach's cut-off scores (37) for EE, DA, and PA is 25, 11, and 16, respectively. According to the scores for these three dimensions, the model then divides burnout into four levels in Table 1. In the current study, reliability measured by Cronbach's alpha coefficient for the whole burnout, EE, DA, and reduced PA in this study were 0.928, 0.985, 0.938, and 0.900, respectively.

**Table 1:** The cut-off scores for burnout three dimensions.

**Variables classification**	**Burnout degree**	**Range scores**
Emotional exhaustion	Mild	0–10
	Moderate	11–15
	Severe	16–30
Depersonalization	Mild	0–9
	Moderate	9–12
	Severe	13–24
Reduced personal accomplishment	Mild	0–18
	Moderate	19–22
	Severe	22–36
Burnout	No burnout	EE <25, DA <11, and PA <16
	Mild burnout	one of the three scores is greater than or equal to cut-off (EE, DA, and reduced PA are 25, 11, and 16)
	Moderate burnout	two of the three scores are greater than or equal to cut-off (EE, DA, and reduced PA are 25, 11, and 16)
	Severe burnout	EE>25, DA>11, and PA> 16

### Statistical Analysis

Statistical significance was considered as a two-tailed *p*-value < 0.05. All analyses were conducted using SPSS version 22.0 (IBM, BM SPSS Statistics for Windows). Demographic factors and job characteristics had been defined as independent variables, and total burnout had been defined as a dependent variable. Univariate and multivariate logistic regression analyses as the main statistical analysis methods were performed in the current study.

## Results

### Demographic Information for Samples

The age of the participants ranged from 20 to 55 years. Most (77.00%) participants had bachelor's degree and above. Of the 1,133 sampled nurses, 45.1% of them were unmarried, approximately 53.3% were married, and 1.6% were divorced or had loss of a spouse. Approximately 27.7% of them belonged to the primary professional category. Furthermore, a total of 529 nurses (46.7%) worked less than 6 years. Moreover, 74.8 and 32.2% of them experienced night-shifts work and worked 40 hours/week, respectively. Moreover, 27.19% had a monthly income within 2,001–5,000 RMB, as shown in Table 2.

**Table 2:** The list of demographic and job characteristics of the participants.

**Demographic Characteristics**	***N***	***%***	**Job Characteristics**	***N***	***%***
Age			Professional title		
20–29	647	57.1	contrast = Primary: nurse	314	27.7
30–39	387	34.2	Primary: senior nurse	506	44.7
40–49	82	7.2	Medium: nurse-in-charge	272	24.0
50+	17	1.5	High: associate professor of nursing/professor of nursing	41	3.6
Marital status			Hospital level		
Marital status: unmarried	511	45.1	tertiary hospitals	160	14.1
Marital status: married	604	53.3	non-tertiary hospitals	973	85.9
Marital status: divorce or loss of spouse	18	1.6	hours/week		
Education level			<40hours	768	67.8
Education level: technical secondary school	13	1.1	>40hours	365	32.2
Education level: college degree	247	21.8	Job tenure		
Education level: bachelor	816	72.0	1–3 years	365	32.2
Education level: master and above	57	5.0	4–5 years	164	14.5
Parental status			6–10 years	316	27.9
no child	655	57.9	11–20years	212	18.7
0–5years	308	27.2	21 and above years	76	6.7
6–18 years	125	11.0	Monthly salary income (RMB)		
19 +	44	3.9	3000 or below	84	7.4
			3001–5000	224	19.8
			5001–7000	292	25.8
			7001–9000	307	27.1
			9001 or above=5	226	19.9
			Night shift		
			no	285	25.2
			yes	848	74.8

*Senior nurse: Conduct clinical nursing work and technical operations and teach nursing students. Nurse-in-charge: Intermediate professional title, inspect, and supervise the clinical work of nurses. Associate professor of nursing/Professor of nursing: Senior professional title, manage clinical staff, and promote professional development*.

### The Incidence of Burnout and the Three Dimensions

The status of burnout of female nurses is shown in Table 3. Among 1,133 participating Chinese female nurses, a total of 782 reported experiencing varying degrees of burnout over the 2 weeks. The overall prevalence of all degrees of burnout was 60.20%, and the breakdown according to severity is as follows: 451 (39.8 %) mild, 163 (14.4%) moderate, and 68 (6.0%) severe burnout.

**Table 3:** Values are numbers (percentages) of respondents regarding burnout (*n* = 1,133).

**Variables classification**	**Burnout degree**	***N***	***%***	***M***	***SD***
Emotional exhaustion	Mild	547	48.3	12.63	7.35
	Moderate	288	25.4		
	Severe	298	26.3		
Depersonalization	Mild	757	66.8	7.54	6.02
	Moderate	190	16.8		
	Severe	186	16.4		
Reduced personal accomplishment	Mild	779	68.8	14.21	7.29
	Moderate	228	20.1		
	Severe	126	11.1		
Burnout	No burnout	451	39.8	34.39	16.85
	Mild burnout	451	39.8		
	Moderate burnout	163	14.4		
	Severe burnout	68	6.0		

### Total Burnout-Univariate Analysis

The analysis of the factors influencing burnout of Chinese female nurse is shown in Tables 4, 5. One of the classification variables was intentionally set as a “dummy” variable. Univariate logistic regression analysis of the development dataset included in the equation was as follows: (1) burnout as dependent variable (never = 0, exist = 1); (2) age of nurse (20–29 = 1, 30–39 = 2, 40–49 = 3, 50+ = 4); (3) marital status (unmarried = 1, married = 2, divorced or loss of a spouse = 3); (4) education level (technical secondary school= 1, college degree = 2, bachelors = 3, master degree, or above = 4); (5) parental status was divided into four categories (no child = 1, 0–5years = 2, 6–18 years = 3, 19, and above years = 4). (6) Professional title (primary: nurse = 1, primary: senior nurse = 2, medium: nurse-in-charge = 3, high: associate professor of nursing/professor of nursing = 4); (7) hospital level (tertiary hospitals = 1, non-tertiary hospitals = 2); and (8) hours/week (≤ 40 hours/week = 1, >40 hours/week = 2). (9) Job tenure (1–3 years = 1, 4–5 years = 2, 6–10 years = 3, 11–20 years = 4, 21, and above years = 5); (10) monthly salary income (RMB) (3,000 or below = 1, 3,001–5,000 = 2, 5,001–7,000 = 3, 7,001–9,000 = 4, 9,001 or above = 5); (11) night shifts (no = 1, yes = 2). According to our results, most female nurses (61.20%) exhibited varying degrees of burnout. Compared to female nurses aged 20–29 years old, female nurses aged 40–49 were not as prone to burnout. Age (*Wald*χ^2^ = 14.562, *P* < 0.01, OR <1), parental status (*Wald*χ^2^ = 17.688, *P* < 0.05, OR <1), professional title (*Wald*χ^2^ = 12.748, *P* < 0.01, OR <1), working hours (*Wald*χ^2^= 4.466, *P* < 0.05, OR > 1), job tenure (*Wald*χ^2^ = 28.826, *P* < 0.05, OR > 1), monthly salary income (RMB) (*Wald*χ^2^ = 13.692, *P* < 0.05, OR > 1), and night shifts (*Wald*χ^2^ = 5.341, *P* < 0.05, OR > 1) were the factors associated with burnout. These seven factors were then entered into the multivariate logistic regression model.

**Table 4:** Univariate logistic regression analysis of respondents (*n* = 1,133).

**Demographic characteristics**	***B***	***S.E*.**	***Wald***	***Df***	***P***	***OR***	***LLUI***	***ULCI***
Age (contrast =20–29)			14.562	3	0.002			
30–39	−0.027	0.132	0.041	1	0.839	0.973	0.751	1.261
40–49	−0.891	0.239	13.844	1	0.000	0.410	0.257	0.656
50+	−0.377	0.493	0.587	1	0.444	0.686	0.261	1.801
Marital status (contrast =unmarried)			4.829	2	0.089			
Marital status: married	−0.214	0.123	3.004	1	0.083	0.807	0.634	1.028
Marital status: divorce or loss of spouse	−0.764	0.483	2.503	1	0.114	0.466	0.181	1.200
Education level (contrast = technical secondary school)			2.574	3	0.462			
Education level: college degree	−0.492	0.615	0.642	1	0.423	0.611	0.183	2.038
Education level: bachelor	−0.398	0.605	0.433	1	0.510	0.671	0.205	2.199
Education level: master and above	−0.038	0.665	0.003	1	0.955	0.963	0.262	3.546
Parental status (contrast = no child)			17.688	3	0.001			
0–5years	−0.067	0.142	0.222	1	0.637	0.935	0.707	1.236
6–18 years	−0.624	0.196	10.069	1	0.002	0.536	0.365	0.788
19 +	−0.968	0.322	9.030	1	0.003	0.380	0.202	0.714

**Table 5:** Univariate logistic regression analysis of respondents (*n* = 1,133).

**Job characteristics**	***B***	***S.E*.**	***Wald***	***Df***	***P***	***OR***	***LLUI***	***ULCI***
Professional title (contrast = Primary: nurse)			12.748	3	0.005			
Primary: senior nurse	0.172	0.148	1.350	1	0.245	1.188	0.889	1.587
Medium: nurse-in-charge	−0.177	0.168	1.111	1	0.292	0.838	0.603	1.164
High: associate professor of nursing/professor of nursing	−0.860	0.340	6.383	1	0.012	0.423	0.217	0.825
Hospital level (contrast =tertiary hospitals)								
non-tertiary hospitals	0.335	0.180	3.449	1	0.063	1.397	0.982	1.989
hours/week (contrast= <40hours)								
>40hours	0.278	0.132	4.466	1	0.035	1.321	1.020	1.710
job tenure (contrast =1–3 years)			28.826	4	0.000			
4–5 years	0.005	0.195	0.001	1	0.978	1.005	0.686	1.474
6–10 years	0.111	0.161	0.475	1	0.491	1.117	0.815	1.531
11–20years	−0.431	0.175	6.054	1	0.014	0.650	0.461	0.916
21 and above years	−1.141	0.263	18.789	1	0.001	0.320	0.191	0.535
Monthly salary income (RMB) (contrast =3000 or below)			13.692	4	0.008			
3001–5000	−0.179	0.272	0.433	1	0.511	0.836	0.491	1.425
5001–7000	−0.110	0.264	0.174	1	0.677	0.896	0.534	1.503
7001–9000	−0.531	0.260	4.167	1	0.041	0.588	0.353	0.979
9001 or above=5	−0.605	0.269	5.064	1	0.024	0.546	0.322	0.925
Night shift (contrast =no)								
yes	0.320	0.138	5.341	1	0.021	1.377	1.050	1.806

### Total Burnout-Multivariate Analysis

Further screening of the seven factors influencing burnout of female nurses is shown in Table 6. We analyzed the four factors selected by the univariate logistic regression analysis using multivariate factor logistic regression analysis. According to Table 4, the result of the significance test of the whole regression model is *X*^2^ = 60.991 (*P* = 0.001*, P* < 0.01), while the result of the Hosmer-Lemeshow test is 2.132 (*P* = 0.459, *P* > 0.05); therefore, these regression models were optimal. As shown in Table 4, the results of the tests concerning the variables of job tenure (*Wald*χ^2^ = 14.828, *P* < 0.05, OR <1), night shift (*Wald*χ^2^ = 3.821, *P* < 0.01, OR > 1), and monthly salary income (RMB) (*Wald*χ^2^ = 12.460, *P* < 0.01, OR <1) were significant, suggesting that these factors are the significant predictors of burnout among female nurses.

**Table 6:** Multivariate logistic regression analysis of respondents (n = 1,133).

**Variables**	**B**	**S.E**.	**Wald**	**Df**	**P**	**OR**	**LLUI**	**ULCI**	**Correlation strength**
Age (contrast = 20–29)			7.233	3	0.065				
30–39	0.551	0.237	5.415	1	0.020	1.735	1.091	2.760	
40–49	0.829	0.488	2.884	1	0.089	2.290	0.880	5.958	
50+	1.590	0.807	3.879	1	0.049	4.905	1.008	23.871	
Parental status (contrast = no child)			1.871	3	0.600				
0–5years	−0.087	0.180	0.233	1	0.629	0.917	0.645	1.304	
6–18 years	−0.359	0.276	1.686	1	0.194	0.699	0.407	1.200	
19 +	−0.423	0.540	0.612	1	0.434	0.655	0.227	1.889	
Job tenure (contrast =1–3 years)			14.828	4	0.005				
4–5 years	−0.135	0.225	0.362	1	0.547	0.873	0.562	1.357	
6–10 years	−0.323	0.247	1.712	1	0.191	0.724	0.447	1.174	
11–20years	−1.043	0.340	9.398	1	0.002	0.352	0.181	0.686	
21 and above years	−1.989	0.597	11.099	1	0.001	0.137	0.042	0.441	
Professional title (contrast =Primary: nurse)			6.135	3	0.105				
Primary: senior nurse	0.485	0.205	5.600	1	0.018	1.624	1.087	2.427	
Medium: nurse-in-charge	0.609	0.291	4.382	1	0.036	1.838	1.040	3.251	*Cox-Snell R_2 =_*0.052 *Nagelkerke R_2 =_*0.071
High: associate professor of nursing/professor of nursing	0.557	0.483	1.332	1	0.248	1.746	0.678	4.496	
Monthly salary income (RMB) (contrast =3000 or below)			12.460	4	0.014				
3001–5000	−0.220	0.282	0.606	1	0.436	0.803	0.462	1.396	
5001–7000	−0.179	0.284	0.399	1	0.528	0.836	0.479	1.458	
7001–9000	−0.612	0.285	4.609	1	0.032	0.542	0.310	0.948	
9001 or above=5	−0.677	0.301	5.045	1	0.025	0.508	0.281	0.917	
Hours/week (contrast= <40 hours)	0.116	0.161	0.518	1	0.472	1.123	0.819	1.541	
Night shift (contrast =no)	0.267	0.136	3.821	1	0.050	1.306	0.999	1.706	
Constant	−0.586	0.382	2.355	1	0.125	0.557			

*Overall model fit test X2 = 60.991 Hosmer-Lemeshow = 7.743*.

## Discussion

### Prevalence of Burnout Among Chinese Female Nurses During the Controlled COVID-19 Period

The burnout incidence in female nurses during the controlled COVID-19 period was 60.20%, with a breakdown in severity as follows: 451 (39.8 %) mild, 163 (14.4%) moderate, and 68 (6.0%) severe burnout, which was slightly lower than the initial stage of COVID-19 outbreak and slightly lower than that of predating COVID-19 ones (13). The current study on burnout in Chinese female nurses reported that 26.3% with high EE, 16.4% with high levels of DA, and 11.1% with low levels of personal achievement respectively among the participates. During the period of the COVID-19 outbreak, the anti-epidemic efforts of female nurses were positively evaluated by the public and media in China, which heartened the sense of achievement, career identity, and high-level self-recognition of female nurses, thus showing a decreased EE and less DA but more PA (38). Compared with Chinese doctors (21, 39), the overall prevalence of burnout of female nurses was lower involved less EE and less DA but more personal accomplishment. The study supports the view that burnout is still a common prevalence and worrying phenomenon in female nursing. In China, due to the increased contradictions between nursing care and expectation of patients (40), the exceeding work-family conflicts and work stress have been increasing especially in female nurses (41), resulting in a variety of potentially fatigued or hazardous circumstances for female nurses. Therefore, strategies, such as increasing job resources (42), designedly allocated to female nurses and establishing a supportive work environment (43) and organizational culture should be encouraged to prevent burnout among female nurses in China.

### Risk Factors Influencing Burnout of Chinese Female Nurse

A previous study showed that age, gender, marital status, parental status, and professional title affected the incidence of burnout of female nurses (44). However, our study found that job characteristics, such as job tenure, monthly salary income, and night shift, were closely linked to the level of burnout, rather than age, marital status, and other demographic characteristics. The current study further confirmed that female nurses with shorter job tenure showed a greater vulnerability toward suffering burnout than those with longer job tenure. Compared to broad experienced female nurses, new female nurses may initially feel inadequately prepared for their occupational role, especially at the early stages of a career that often be known as the most stressful, frustrating, discouraging, and disillusioning stage for individual employees, which is also the process of ongoing professional role adaptation and identity crisis (45, 46). For female nurses with shorter job tenure, the experience of transition from school-based experience to professional practice can be stressful compounded by entrenched systemic issues, such as working irregular hours, rotating shifts, and under staffing (45). Being shorter job tenure, those nurses do not yet possess solid interpersonal and workplace relationships that are conducive to destroy intrapersonal psychological capital against burnout (47). Therefore, much more attention should be paid to that new female nurse is suffering from high-level burnout.

A previous study proved that inadequate sleep was associated with increasing burnout in shift working occupations (48). The current findings showed that working on night shifts is also one risk factor for female nurses suffering burnout rather than their weekly work hours. As an inevitable work type for providing in-patient nursing care, night shifts for female nurses were associated with more reports of heavy burnout symptoms (49, 50). A nurse working irregular shifts was more likely to lead to inadequate sleep (51), irregular habits and lifestyles (52), and change in sleep blood pressure (50), which in turn may explain the increased overall burnout risk among female nurses compared with those working daily (50). Inadequate sleep caused by irregular shifts might destroy accessibility and ability of female nurses to recover from fatigue caused by heavy duty and workplace demands, potentially resulting in the heightened burnout risk in those reporting irregular shifts (48). Unlike the western countries, the shortage of nurses in China is more serious due to the huge China population base and inefficient health service systems (53), thereby the nurses have been encountered heavier workload and more frequent irregular shifts, resulting in a range of serious consequences, such as sleep disorder and physical damage for a long time (54). Although shift work is unavoidable for a nursing career, it is valuable for a manager that optimizing shift arrangement in relation to control over overtime should be explored as one of the key intervention to prevent the high-level burnout among female nurses, thereby concentrating on improved health and wellness for female nurses and in turn enhanced patient care quality.

Female nurses with lower monthly salary income were more likely to report severe burnout than that those with higher monthly salary income, which seems to be at coherent with previous findings (55). To some extent, low salary is one of the key predictor for a high risk of burnout among female nurses might as a result of perceived disproportion between job resources and demands (56, 57). Perceived unreasonable revenue as an important determinant factor for female nurses is likely to lead to decreased motivation and job satisfaction and then resulting in high-level burnout (58), which also reversely interpreted that the participating female nurses in this study who reported high-level salary tend to feel less emotionally exhausted and less depersonalized but more personally accomplished. Besides, another reason might be that the general judgment indicator of PA of female nurses was determined by the money their earn (59). Previous studies revealed that Chinese nurses are dissatisfied with their salary (60), working environment, and nurse-patient relationships. Moreover, compared to the pre-COVID-19 epidemic stage, work pattern and workflow of nurse are completely different from the usual during controlling the COVID-19 epidemic. According to the current study, the finding suggested that 32.2% of the participants had hours/week more than 40 hours/week, indicating that nearly a third of the participants had complained the long working hours during the controlled COVID-19 period. In this stage, Chinese nurses are facing various huge challenges, such as increased the long working hours, extra effort, heavily workload, stress, new risk, and so on (20, 53). Except for front-line wards nurses, the majority of Chinese nurse's salary did not increase due to decreased hospital economic benefit during a fight against the COVID-19 epidemic period, which contributes to the effort-reward imbalance at work being more serious. Therefore, it is important for nurse administrators and managers to enable female nurses to alleviate work-related burnout symptoms through matching reasonably economic, physical, and emotional resources (61). The last evidence from The Dresden Burnout Study (DBS) is a 12-year longitudinal cohort study that aims to provide a description of the burnout syndrome on the basis of time and symptom criteria with a special focus on the search for biomarkers (62) indicated that providing work environments where high efforts are always linked with high rewards, such as economic, physical, and emotional resources, have to be considered an important issue for avoiding burnout (63).

### Limitations

In the current study, the findings need to be discussed with respect to certain limitations. First, data were collected through an online survey, which is prone to lead to response bias due to social desirability or negative effect. Second, although these efforts had been tried to ensure a representative sample and although there were no significant differences across data collection regions, we must acknowledge that the effective response rate was not ideal. Third, a cross-sectional design cannot determine a cause-effect relationship among the studied variables. Further experimental design is needed to test the cause-effect relationships across different cultural contexts. Forth, due to the substantial existence of income differences between the eastern and western areas in China, the categories of monthly salary incomes in our study only represent ordinal variables, which cannot explain the impact of the differences of real income between regions on burnout. Fifth, the multicollinearity problem is a substantial existence in our study, which potentially leads to skewed or misleading results when the current study attempts to determine how well each independent variable can be used most effectively to predict or understand the dependent variable in a statistical model. Last, burnout among nurses is constantly changing during the epidemic, but our study only focuses on a single time point in China from September to October 2020.

## Conclusions

This study revealed the prevalence of burnout syndrome among female nurses (60.02%) during the controlled COVID-19 period. Female nurses reported that more of them were likely to experience higher incidence and more severity of EE than that of DA and reduced PA. Given the growing number of female nurses entering the field of healthcare service, much attention should be paid to their EE dimension. Furthermore, the interventions and prevention measures direct at female burnout should be strengthened and improved, especially during the controlled COVID-19 period, further rather than that be ignored. Females bring many innate qualities to their work as nurse, which affects burnout due to differentiated socio-demographic characters. Socio-demographic factors of female nurses, such as job tenure, salary income, and night shifts, accounted for only a small proportion of variance in the burnout of nurses in Chinese nurses during the controlled COVID-19 period. Shorter-tenure female nurses experienced a much higher level of burnout symptoms than that those with longer job tenure. Female nurses who worked at night shifts and had lower monthly salary tend to exhibit an increasing high risk of burnout. The current study suggests that hospital administrators and decision-makers should provide positive psychological support to avert and alleviate the burnout symptoms of female nurses during the controlled COVID-19 period, especially focusing on nurses who are younger, have lower salary, and have night shift.

## Data Availability Statement

The datasets used and/or analyzed during this study are available from the corresponding author on reasonable request from hydsuntao@126.com.

## Ethics Statement

The study was conducted according to the guidelines of the Declaration of Helsinki, and approved by the Institutional Review Board of Harbin Medical University (ECHMU). Due to the anonymous survey approach, written informed consent could not be obtained. A verbal informed consent form was included at the beginning of the questionnaire. And the ECHMU had approved verbal consent for this study. Completing the questionnaire regarded as a verbal consent was therefore considered ‘informed consent' for participation in the survey. A verbal informed consent has been obtained from the participants to publish this paper.

## Author Contributions

L-LZ, S-EZ, and TS conceived and designed the experiments. JL, H-NW, LL, Y-FG, J-JZ, and Z-HB performed the experiments. S-EZ and BL analyzed the data. JL, H-NW, and Y-FG contributed reagents, materials, analysis tools. L-LZ and S-EZ wrote the paper. BL and TS approved the final manuscript for publication. All authors contributed to the article and approved the submitted version.

## Conflict of Interest

The authors declare that the research was conducted in the absence of any commercial or financial relationships that could be construed as a potential conflict of interest.

## Publisher's Note

All claims expressed in this article are solely those of the authors and do not necessarily represent those of their affiliated organizations, or those of the publisher, the editors and the reviewers. Any product that may be evaluated in this article, or claim that may be made by its manufacturer, is not guaranteed or endorsed by the publisher.
